# TCRscape: a single-cell multi-omic TCR profiling toolkit

**DOI:** 10.3389/fbinf.2025.1641491

**Published:** 2025-09-05

**Authors:** Roman Perik-Zavodskii, Olga Perik-Zavodskaia, Marina Volynets, Saleh Alrhmoun, Sergey Sennikov

**Affiliations:** Laboratory of Molecular Immunology, Federal State Budgetary Scientific Institution Research Institute of Fundamental and Clinical Immunology, Novosibirsk, Russia

**Keywords:** scRNA-seq, scTCR-seq, TCR, T-cell receptor, clonotype, CDR3, TCR T-cells

## Abstract

**Introduction:**

Single-cell multi-omics has transformed T-cell biology by enabling the simultaneous analysis of T-cell receptor (TCR) sequences, transcriptomes, and surface proteins at the resolution of individual cells. These capabilities are critical for identifying antigen-specific T-cells and accelerating the development of TCR-based immunotherapies.

**Methods:**

Here, we introduce TCRscape, an open-source Python 3 tool designed for high-resolution T-cell receptor clonotype discovery and quantification, optimized for BD Rhapsody™ single-cell multi-omics data.

**Results:**

TCRscape integrates full-length TCR sequence data with gene expression profiles and surface protein expression to enable multimodal clustering of αβ and γδ T-cell populations. It also outputs Seurat-compatible matrices, facilitating downstream visualization and analysis in standard single-cell analysis environments.

**Discussion:**

By bridging clonotype detection with immune cell transcriptome, proteome, and antigen specificity profiling, TCRscape supports rapid identification of dominant T-cell clones and their functional phenotypes, offering a powerful resource for immune monitoring and TCR-engineered therapeutic development. TCRscape can be found at https://github.com/Perik-Zavodskii/TCRscape/.

## 1 Introduction

T-cells are specialized white blood cells that play a central role in the adaptive immune system, which helps the body fight off specific pathogens and abnormal cells like cancer cells. These cells detect unique molecules, known as antigens, from bacteria, viruses, or cancerous cells through a process called antigen presentation. Antigen-presenting cells (APCs), such as dendritic cells, macrophages, and B cells, break down antigens and display them on their surface using major histocompatibility complex (MHC) molecules, allowing T-cells to recognize and respond to threats effectively (Jung and Alt, 2004).

Each T-cell has a unique T-cell receptor (TCR), generated through a genetic process called V (D)J recombination. In this process, gene segments, Variable (V), Diversity (D), and Joining (J), are randomly shuffled and combined to create diverse TCR sequences. For most T-cells, TCRs consist of alpha (TCRα) and beta (TCRβ) chains, while a smaller subset, known as gamma delta T-cells, has TCRs made of gamma (TCRγ) and delta (TCRδ) chains. Gamma delta T-cells are unique because they can recognize a broader range of antigens, including non-peptide molecules, and often respond to stress signals from infected or cancerous cells. The TCR’s complementarity-determining region 3 (CDR3), shaped by V(D)J recombination, is the most variable part and directly binds to the antigen-MHC complex, determining the T-cell’s specificity (Dash et al., 2017).

**FIGURE 1:**
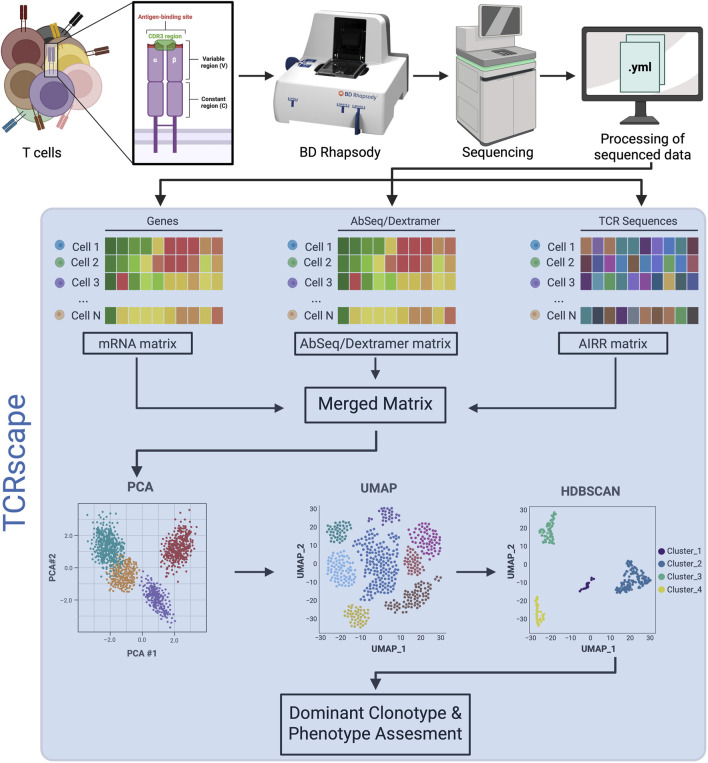
Overview of the sequential steps in TCR repertoire analysis using the BD Rhapsody platform. The blue-shaded area represents the TCRscape analysis pipeline.

Upon antigen presentation, T-cells with TCRs that recognize the antigen-MHC complex undergo clonal expansion, rapidly proliferating to produce a population of identical cells with high-affinity TCRs. This process amplifies specific T-cell clones that form TCR clonotypes, mostly defined by the unique pairing of their alpha and beta chain complementarity-determining region 3 (CDR3) sequences, enabling targeted immune responses against infections or malignancies (Pai and Satpathy, 2021). TCR clonotypes represent the molecular identity of an individual T-cell’s antigen receptor and serve as a stable fingerprint of clonal lineage and antigen-driven selection. Clonotypes are typically defined by the nucleotide or amino acid sequences of the CDR3 from both the chains of the T-cell receptor, which collectively mediate specific recognition of peptide-MHC complexes (Figure 1) (Pai and Satpathy, 2021; Glanville et al., 2017). Identifying and quantifying TCR clonotypes enables researchers to trace clonal expansions, monitor immune responses, and discover public or private T-cell signatures associated with disease, vaccination, or therapy response. In the context of TCR gene therapy, dominant therapeutic clonotypes can be cloned and inserted into viral vectors, such as HIV-1-based lentiviruses (Perro et al., 2010) or MMLV retroviruses (Alsalloum et al., 2023), to generate engineered T-cells for adoptive transfer. Accurate and scalable detection of these clonotypes is thus fundamental for mechanistic immunology and therapeutic development.

Historically, T-cell receptor sequencing relied on bulk RNA sequencing, a method that extracts and sequences RNA from a large pool of T-cells simultaneously, averaging their genetic signals. This approach, while cost-effective and capable of detecting abundant TCR sequences, mixes RNA from different T-cells, making it impossible to preserve the critical pairing between TCRα/TCRβ or TCRγ/TCRδ chains that defines a T-cell’s unique antigen specificity (Rosati et al., 2017). The advent of single-cell RNA sequencing (scRNA-seq) transformed TCR genomics, making single-cell T-cell repertoire analysis a cornerstone of immunology due to its unparalleled ability to resolve the diversity, specificity, and functional state of T-cells (Pai and Satpathy, 2021). Modern single-cell multi-omics platforms, like 10x Chromium and BD Rhapsody, support high-resolution TCR repertoire analysis, with BD Rhapsody enabling full-length TCR sequencing (V, D, J, and constant regions) (Gao et al., 2020) and 10x Chromium capturing partial V(D)J sequences due to short-read sequencing (Pai and Satpathy, 2021; Byrne et al., 2024). These platforms enable precise TCR chain pairing through cell barcoding, along with parallel transcriptome and proteome profiling of individual T-cells (hence the term multi-omic). When combined with barcode-based MHC-multimer technologies, such as dCODE Dextramer (Ulbrich et al., 2023) (for BD Rhapsody and 10X Genomics Chromium) and BEAM (Shahi et al., 2023) (10X Genomics Chromium), they allow direct inference of antigen specificity. This integration accelerates the development of personalized TCR-based therapies for oncology and infectious diseases. Tracking clonotypes enables researchers to monitor immune responses and develop therapies targeting tumor antigens like MAGE-A3 or mutant p53, opening new frontiers in immunology (Hsiue et al., 2021; Robbins et al., 2013; Sennikov et al., 2024; Goh et al., 2011).

(Gupta et al., 2022; Pai and Satpathy, 2021; Byrne et al., 2024) (Shugay et al., 2015) (GitHub immunomind/immunarch, 2020) (Gao et al., 2020) (Iqbal et al., 2020) Single-cell multiomic analysis has revolutionized TCR analysis, but platforms face wet lab and computational challenges. The 10X Chromium platform, paired with its free Loupe VDJ Browser (Gupta et al., 2022), offers user-friendly visualization of clonotypes and CDR3 sequences from 5′ single-cell immune profiling data. Loupe V(D)J Browser offers intuitive plots (e.g., clonotype distribution, V/J gene usage) and supports paired TCRα/β analysis, but it is tightly integrated with 10x Genomics’ Cell Ranger pipeline, limiting its use to 10x data formats, combined with the partial-length V(D)J sequences produced by the 10x Chromium 5′assay due to short-read sequencing, constrains its broader utility in immune repertoire studies (Pai and Satpathy, 2021; Byrne et al., 2024). Additionally, it lacks advanced customization for downstream analyses, requiring users to export data to other tools like VDJtools (Shugay et al., 2015) or Immunarch (GitHub immunomind/immunarch, 2020) for deeper insights. In contrast, the BD Rhapsody™ platform, which supports targeted scRNA-seq with potential for full-length TCR sequencing (Gao et al., 2020), relies on SeqGeq (Iqbal et al., 2020), a commercial software with a graphical interface for analyzing targeted single-cell RNA-seq data. SeqGeq supports basic TCR clonotype visualization but has limited functionality for full-length TCR clonotype quantification and struggles with compatibility for common downstream analysis frameworks, often requiring custom scripts for comprehensive analysis.

To fill this gap, we developed TCRscape, our open-source Python 3 tool, that enables high-resolution TCR clonotype detection, quantification, and multimodal integration for standardized single-cell sequencing matrices from BD Rhapsody™. Unlike Loupe V(D)J Browser’s platform-specific design or SeqGeq’s commercial and limited functionality, TCRscape provides a user-friendly, code-accessible pipeline for researchers with minimal programming expertise and outputs clonotype data in a structure that is directly compatible with popular single-cell analysis libraries such as Seurat (Hao et al., 2024) and Scanpy (Wolf et al., 2018). By leveraging gene and protein expression and V(D)J segment usage, TCRscape enables clonotype clustering and downstream applications, including UMAP-based visualization, clonotype enrichment, and candidate TCR discovery for therapeutic development (Figure 1).

## 2 Materials and methods

### 2.1 Data import into TCRscape

TCRscape imports BD Rhapsody™ multi-omic expression matrices in the standard 10X Genomics-like Feature-Matrix-Barcode format via the *ReadRhapsody()* function. It also imports the corresponding Adaptive Immune Receptor Repertoire (AIRR) matrix (usually named “Dominant_Contigs_AIRR.tsv”) as Pandas (The pandas development team, 2024) data frames. Each gene expression matrix is manually assigned its Sample Tag to track sample provenance. In this paper, we used a dataset that represents CD8^+^ T-cells before (“Pre-treatment”, n = 2) and after the stimulation with the human papillomavirus (HPV) antigens (“Post-treatment”, n = 2) presented to them by dendritic cells (DCs) loaded with the soluble HPV antigen peptides.

### 2.2 Data normalization

To normalize gene expression data, TCRscape merges samples via the *MergeRhapsody()* function, and then applies UMI counts data normalization (factor = 10.000), followed by log2 transformation with a pseudocount using NumPy (Harris et al., 2020) via the *LogNormalize()* function. This produces a normalized matrix suitable for downstream clustering and feature extraction.

### 2.3 T-cell gating

TCRscape allows for automatic gating of T-cells via the *GateTcells()* function and their subsets–CD4 and CD8 T-cells via the *GateCD4()* and *GateCD8()* functions, respectively. Manual T-cell gating can also be performed at this stage (i.e., dCODE Dextramer positive T-cell selection) and visualized via matplotlib (The Matplotlib Development Team, 2024). For high-dimensional datasets, automatic selection using dimensionality reduction (e.g., UMAP (McInnes et al., 2018)) and clustering (e.g., HDBSCAN (McInnes et al., 2017)) is supported.

### 2.4 T-cell clonotype detection and quantification

As a preprocessing step, immunoglobulin sequences are removed from the AIRR matrix. Incomplete TCR constant regions are restored, such as reconstructing TRBC1 for beta chains, and pseudo-translated 5′-UTR regions are trimmed to the first methionine following a stop codon. TCR amino-acid sequences are separated by chain type (TRA, TRB, TRG, TRD), tagged, and then merged by cell index to form clonotype contigs (e.g., TRA_aa___TRB_aa). Only paired chains are retained. Alpha-beta and gamma-delta clonotypes are handled separately. Clonotype counts are computed as the number of cells per unique contig via the *CountClonotypes()* function.

### 2.5 T-cell CDR3-type detection and quantification

To identify CDR3-types, TCRscape extracts the CDR3 amino-acid sequences for each chain, tags them, and merges them into CDR3-type contigs per cell (e.g., TRA_CDR3___TRB_CDR3). As with full-length clonotypes, only paired chains are retained. CDR3-type counts are computed as the number of cells per unique CDR3 pair via the *CountCDR3()* function. Visualization also includes a pie chart via the *CDR3Pie()* function.

### 2.6 Multimodal embedding and clustering of T-cells

TCRscape performs multimodal clustering using gene expression and TCR-derived features via its main function, *TCRscape()*. A user-defined list of markers (e.g., *CD4, CD8A, NKG7, FOXP3*, and Sample Tag ID) is subsetted from the normalized gene expression matrix. Clonotype counts are quantified and merged with the cell index. The resulting feature matrix is extended via one-hot encoding using the *get_dummies()* function on categorical variables like Sample Tag ID, clonotype ID, and TCR type (where alpha-beta = 1, gamma-delta = 0).

To improve clonotype-level resolution, TCRscape also includes one-hot encoding of V, D, and J gene segments for TRA, TRB, TRG, and TRD chains. Productive TCR sequences are filtered by UMI count, and for each cell, only paired chains are retained. Alpha-beta and gamma-delta cells are processed separately. For each cell, gene segments are extracted and one-hot encoded using pandas. Only relevant segments for the expressed TCR type are retained. This additional feature layer enables finer clustering of clonotypes that may not have identical amino-acid sequences but share similar V(D)J gene usage, thus capturing convergent recombination patterns and antigen-driven selection.

The final feature matrix is optionally standardized and subjected to PCA for linear dimensionality reduction. A UMAP (McInnes et al., 2018) embedding is computed on the top principal components, followed by density-based clustering using HDBSCAN (McInnes et al., 2017). The results are visualized in UMAP space using matplotlib.

### 2.7 TCRscape output data export to Seurat

TCRscape results can be exported as. csv files for use in Seurat (Hao et al., 2024) or Scanpy (Wolf et al., 2018) (Hao et al., 2024). Each TCRscape data frame is left-joined to the cell index of the expression matrix, and missing values are filled with zeros. This ensures compatibility with Seurat’s *CreateAssayObject()* function.

### 2.8 Post TCRscape data analysis in Seurat

In Seurat, BD Rhapsody™ expression data are loaded using the *Read10X()* function, while the exported TCRscape matrices are loaded as a sparse table and assigned to a new TCR assay via the *CreateAssayObject()* function. Multiple samples can be merged with the *merge()* function, filtered using quality control metrics (e.g., nCount_RNA > 100, nFeature_RNA < 6,000), and normalized using SCTransform V2. Batch effects are corrected via Harmony (Korsunsky et al., 2018). Clustering is performed using the *RunPCA()*, *FindNeighbors()* (or *FindMultiModalNeighbors()* for Seurat WNN), and *RunUMAP()* on the Harmony-corrected or the Seurat WNN reduction data. TCRscape-derived data, such as clonotype frequency or dominant TCR identity, can be visualized using the *FeaturePlot()* function to identify immunologically distinct clusters.

## 3 Results

### 3.1 CDR3-type and clonotype discovery and visualization in TCRscape

**FIGURE 2:**
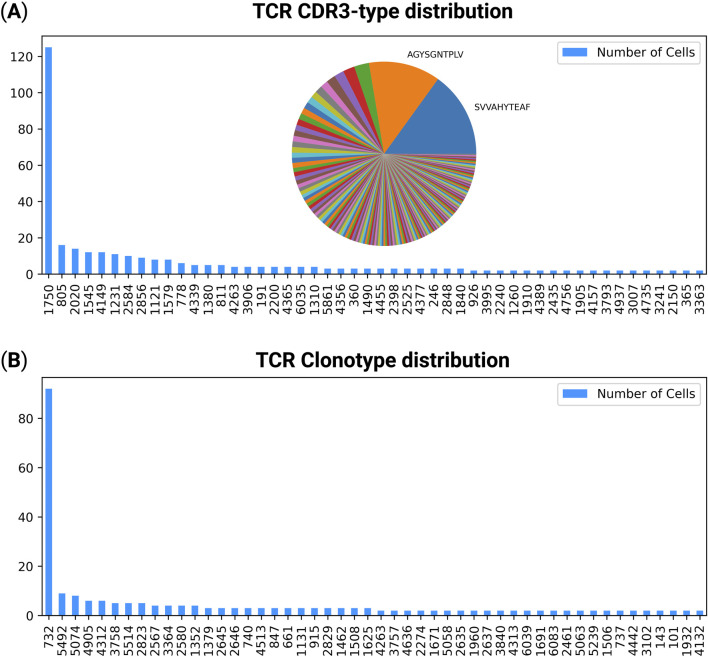
Distribution of T-cell clonotypes and CDR3-types in the post-treatment group. **(A)** CDR3-type distribution across single T-cells. Each bar represents the number of cells sharing a unique CDR3 amino acid sequence, visualized as a bar plot with an embedded pie chart illustrating the relative abundance of the most dominant CDR3-types. **(B)** Clonotype distribution showing the number of cells sharing full-length paired TCR alpha and beta chains. The number under each CDR3-type or clonotype is its automatically assigned hash number.

TCRscape quantifies both CDR3-types and full-length TCR clonotypes across single cells by aggregating the number of cells sharing identical amino-acid sequences. CDR3-types were defined by the combined alpha/beta or gamma-delta chain CDR3 amino acid sequences, while clonotypes were defined as full-length paired alpha/beta or gamma-delta contigs. The resulting frequency distributions for the post-treatment experimental group were visualized using bar plots, with dominant CDR3-types additionally shown in an embedded pie chart for clearer comparison of clonal contributions (Figure 2).

### 3.2 Multimodal T-cell analysis via the usage of full-length TCR clonotypes, V(D)J genes, and hallmark T-cell genes in TCRscape

**FIGURE 3:**
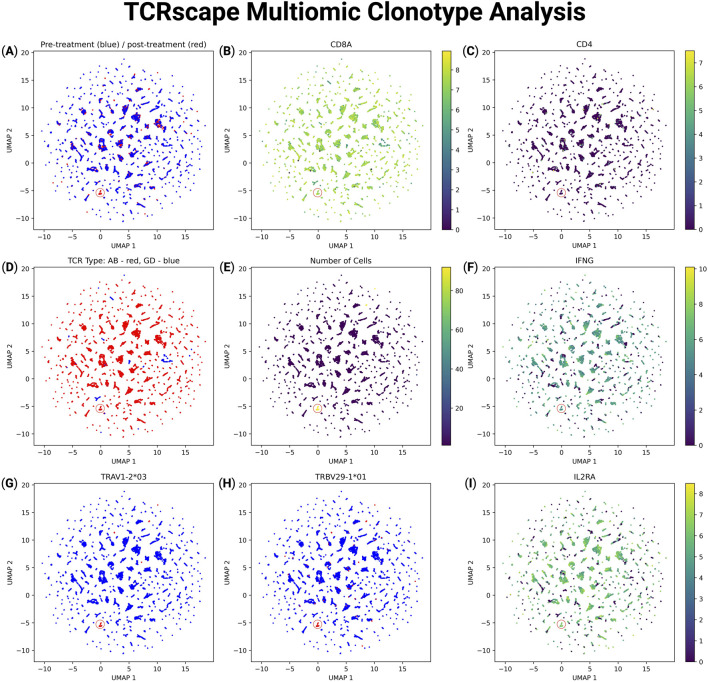
Multimodal UMAP visualization of T-cell features and clonotype data using TCRscape. Each point represents a single T-cell, colored according to the intensity of the given feature. **(A)** UMAP plot reflecting the experimental group: blue–pre-treatment, red–post-treatment, **(B)** UMAP plot reflecting the *CD8A* normalized gene expression, **(C)** UMAP plot reflecting the *CD4* normalized gene expression, **(D)** UMAP plot reflecting the TCR type: blue–gamma-delta TCR, red–alpha-beta TCR, **(E)** UMAP plot reflecting the number of cells per TCR clonotype, **(F)** UMAP plot reflecting the *IFNG* normalized gene expression, **(G)** UMAP plot reflecting the alpha chain V fragment expression of the dominant TCR clonotype, **(H)** UMAP plot reflecting the beta chain V fragment expression of the dominant TCR clonotype, **(I)** UMAP plot reflecting the *IL2RA* (CD25) normalized gene expression.

To explore the transcriptional and clonotypic landscape of T-cells, we projected multimodal data onto UMAP space using features processed by TCRscape (Figure 3). We observed T-cell clustering according to their clonotype data (Figures 3A,E,G,H) with no batch effect observed for the data (Figure 3A). We were also able to observe that there is a dominant clonotype present in the post-treatment group, indicating a potential response to the HPV antigen stimulus (Figures 3A,E). We were also able to identify the TCR type of the dominant clonotype as alpha-beta TCR, as well as characterize the T-cells bearing the dominant clonotype as *CD8* ^+^, *IL2RA* ^+^, *IFNG* ^+^ which is characteristic of T-cells that got activated by an antigen peptide through their TCR (Figures 3B–D,F,I) (Sennikov et al., 2024).

### 3.3 Post TCRscape data analysis in Seurat

**FIGURE 4:**
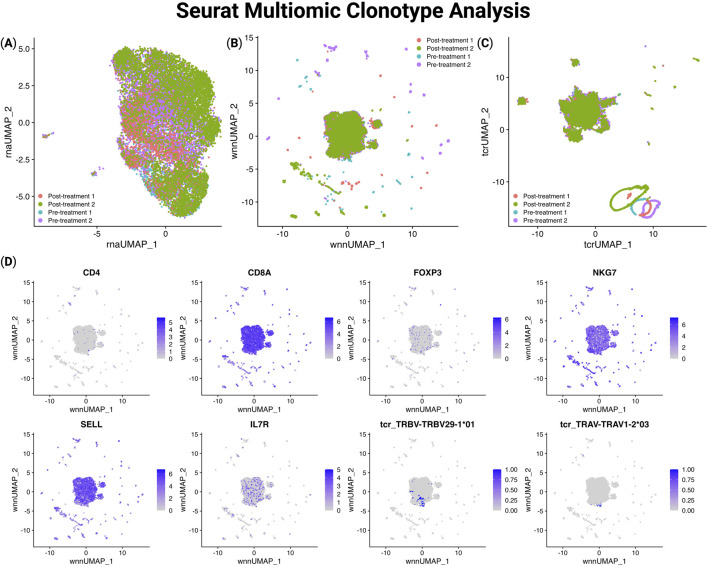
UMAP visualization of gene expression, sample origin, and TCR segment usage in Seurat using TCRscape-integrated data. **(A)** UMAP plot of the gene expression-based dimensionality reduction, **(B)** WNN UMAP plot of the gene expression and TCR data-based dimensionality reduction, **(C)** UMAP plot of the TCR data-based dimensionality reduction, **(D)** WNN UMAP Feature plots of the gene expression and TCR V fragment data.

To demonstrate the importance of the clonotype analysis in TCRscape, we imported the gene expression and TCRscape output data directly into Seurat for downstream visualization and analysis. Importantly, TCRscape-derived outputs such as clonotype identity, TCR type, and V(D)J gene expression were readily incorporated into Seurat using simple import functions. This enables the overlay of clonotypic features on transcriptional space and can help to identify T-cell clonotypes of interest. The seamless integration of TCRscape data into Seurat highlights its utility in multimodal single-cell studies and its potential for extending clonotype analysis in any single-cell multiomic workflow. We used the gene expression and TCR clonotype data to create UMAP and WNN UMAP plots of the data and observed almost no clustering according to T-cell clonotypes, and even the dominant clonotype got merged into a larger cluster (Figure 4).

## 4 Discussion

In this study, we present TCRscape, a powerful and user-friendly tool purpose-built for TCR clonotype discovery and compatible with various single-cell sequencing platforms. Designed to fill a critical analytical gap in TCR and gene expression co-embedding, TCRscape enables seamless detection, quantification, and characterization of both alpha-beta and gamma-delta TCR clonotypes directly from AIRR-compliant matrices, providing a level of flexibility and accessibility that enhances immunological research. Its open-source and Python 3-based architecture makes it an ideal solution for scRNA-seq users, generating outputs that are immediately compatible with popular downstream single-cell analysis platforms like Seurat (Hao et al., 2024) and Scanpy (Wolf et al., 2018), enabling true multimodal integration of TCR identity and transcriptional phenotype.

What distinguishes TCRscape is not only its robustness and simplicity but also its deep analytical capacity. The pipeline goes beyond full-length CDR3-based clonotyping by incorporating one-hot encoding of V(D)J gene segments, enabling the clustering of clonotypes based on shared TCR gene usage, even in the absence of exact sequence identity. This feature is particularly powerful for uncovering convergent recombination events, public clonotypes, and antigen-driven expansions, insights that are critical for vaccine development, immune monitoring, and precision immunotherapy. Moreover, TCRscape allows users to annotate dominant clonotypes, visualize their distribution in transcriptomic space using UMAP and HDBSCAN, and trace their correspondence to functional cell states, such as effector CD8^+^ T-cells or regulatory CD4^+^ T-cells. The export functionality supports a smooth transition to post-processing in Seurat (Wolf et al., 2018), empowering researchers to pair clonotype frequency, phenotypic marker expression, and sample identity within a unified analytical framework. Importantly, TCRscape outputs ready-to-use full-length amino-acid sequences of identified TCRs, which have already been employed in the development of TCR-engineered T-cell therapies targeting tumor antigens (Sennikov et al., 2024; Alrhmoun et al., 2024) and can be used for investigating TCRs associated with tolerance induction in autoimmune contexts. TCRscape also enables clustering of γδ T-cell populations (see Figure 3D), a critical feature for studying their unique roles in innate-like immune responses and cancer immunotherapy, offering potential insights into novel therapeutic targets (Sebestyen et al., 2019; Silva-Santos et al., 2019). Despite requiring moderate Python skills, TCRscape’s intuitive scripts and clear documentation enhance accessibility compared to complex command-line tools.

TCRscape offers distinct advantages over existing TCR analysis tools, including SeqGeq, Loupe VDJ Browser, and other computational pipelines. Unlike SeqGeq, a commercial subscription-based software designed for BD Rhapsody™ multi-omics data, TCRscape is open-source, eliminating cost barriers and enabling greater accessibility for researchers. SeqGeq provides basic TCR clonotype visualization through a graphical interface but lacks robust support for gamma-delta TCRs, full-length clonotype quantification, and compatibility with open-source analysis frameworks like Seurat and Scanpy. Its reliance on proprietary formats and limited scripting flexibility restricts its utility for customized analyses, often requiring users to develop custom scripts for comprehensive TCR studies (Gao et al., 2020). In contrast, TCRscape’s Python-based framework offers extensive scripting flexibility, supports both alpha-beta and gamma-delta TCRs, and generates AIRR-compliant outputs that integrate seamlessly with Seurat and Scanpy, facilitating multimodal analyses.

Loupe VDJ Browser, paired with the 10x Chromium platform, provides user-friendly visualization of clonotypes and CDR3 sequences from 5′ single-cell immune profiling data, offering intuitive plots (e.g., clonotype distribution, V/J gene usage). However, it is tightly integrated with 10x Genomics’ Cell Ranger pipeline, limiting its use to 10x data, and its reliance on partial V(D)J sequences due to short-read sequencing (150–300 bp) constrains its depth for comprehensive TCR repertoire studies (Pai and Satpathy, 2021; Byrne et al., 2024). Additionally, Loupe VDJ Browser lacks advanced analytical features, such as clonotype clustering based on V(D)J gene usage or gamma-delta TCR support, as well as customization for downstream analyses, requiring users to export data to tools like VDJtools (Shugay et al., 2015) or Immunarch (GitHub immunomind/immunarch, 2020) for deeper insights.

Several tools exist for analyzing TCR repertoires from single-cell sequencing data, but each faces significant limitations. Tools like MiXCR (GitHub - milaboratory/mixcr, 2025) and TRUST4 (Song et al., 2021) offer high-accuracy CDR3 and clonotype detection across platforms but demand substantial bioinformatics expertise and complex input preprocessing, hindering accessibility, not to mention that they lack multi-omics integration. Immunarch (GitHub immunomind/immunarch, 2020) provides clonality and diversity analysis in R with moderate accessibility but requires R proficiency and lacks seamless multi-omics integration and support for BD Rhapsody’s full-length sequencing outputs. Decombinator (Thomas et al., 2013) and IMSEQ (GitHub - lkuchenb/imseq, 2020) offer efficient V (D)J gene assignment for TCR sequences but lack robust multi-omics integration and are command-line-based, requiring significant bioinformatics skills. VisTCR (GitHub - qingshanni/VisTCR, 2025) provides user-friendly visualization via an HTML interface but is constrained by limited advanced analytics capabilities and restricted compatibility with non-10x data formats, such as BD Rhapsody, and a lack of built-in multimodal integration for correlating TCR data with transcriptomic or proteomic profiles.

Despite its strengths, TCRscape has areas for future improvement. Currently, it is optimized exclusively for BD Rhapsody data, limiting its compatibility with other single-cell sequencing platforms, such as 10x Genomics, and requiring users to convert or preprocess data from non-Rhapsody sources, limiting its immediate applicability to newer platforms or custom datasets. Additionally, while TCRscape is designed for computational efficiency, its performance with ultra-large datasets (e.g., millions of cells) has not been fully optimized, potentially increasing runtime or memory demands for large-scale studies. Furthermore, TCRscape does not yet include built-in integration with public TCR-antigen databases (e.g., VDJdb, McPAS-TCR), which could enhance its ability to annotate clonotypes with known antigen specificities for immunotherapy applications. Future iterations could address these by expanding compatibility with diverse data formats, optimizing scalability for large datasets, and incorporating direct links to antigen-specificity databases to further streamline translational research.

In summary, TCRscape represents a comprehensive platform for unlocking the full power of single-cell TCR repertoire analysis. By enabling high-resolution clonotype discovery, intuitive visualization, and rich integration with transcriptomic data, TCRscape provides an essential toolkit for modern immunology and translational research. Its open-source nature, advanced analytical features, and practical applications in TCR-engineered therapies and autoimmune research underscore its potential to accelerate discoveries in vaccine development, immune monitoring, and precision immunotherapy.

## Data Availability

Publicly available datasets were analyzed in this study. This data can be found here: https://doi.org/10.5281/zenodo.15280599.
